# Total Laparoscopic Conservative Surgery for an Intramural Ectopic Pregnancy

**DOI:** 10.1155/2010/504062

**Published:** 2010-10-11

**Authors:** Hiroshi Nabeshima, Mitsuo Nishimoto, Hiroki Utsunomiya, Maiko Arai, Tomohisa Ugajin, Yukihiro Terada, Nobuo Yaegashi

**Affiliations:** ^1^Department of Obstetrics and Gynecology, Iwate Prefectural Iwai Hospital, Kozenji, Ichinoseki-city, 029-0192, Japan; ^2^Department of Obstetrics and Gynecology, Tohoku University Hospital, 1-1, Seiryo-machi, Aoba-ku, Sendai-city, Miyagi 980-8574, Japan

## Abstract

A 38-year-old woman, gravida 3, para 1 with a history of a left salpingectomy for an ectopic pregnancy was admitted for treatment of a presumed ectopic pregnancy. Transvaginal sonography revealed an ill-defined gestational sac and fetal heart beat within the fundal myometrium adjacent to the left cornua. Laparoscopy was performed for a suspected left cornual pregnancy or intramural pregnancy. A cystic mass 3 cm in diameter was visible within the fundal myometrium. Total laparoscopic removal of the gestational sac was performed, and the uterus was preserved. Pathologic evaluation of the excised mass demonstrated chorionic villi involving the myometrium. In the literature, only one other case describing the laparoscopic removal of an intramural pregnancy has been reported. However, in the prior report, the patient still required hysterectomy after conservative surgery. Therefore, this is the first report of the successful treatment of an intramural pregnancy exclusively with laparoscopy.

## 1. Introduction

Intramural pregnancy is one of the rarest types of ectopic pregnancy [1]. In the literature, almost all cases have been treated with laparotomy or medication (methotrexate or potassium chloride). There is only one prior report describing the laparoscopic removal of an intramural pregnancy [2]. In that report, conservative surgery was unsuccessful, and the patient went on to have a hysterectomy. We report the first case of an intramural pregnancy treated exclusively with laparoscopy.

## 2. Case

**Figure 1:**
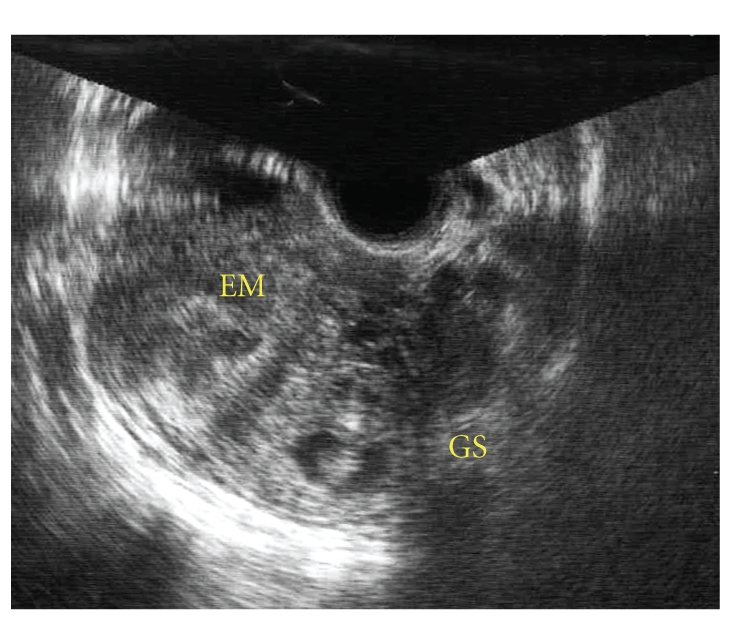
Preoperative ultrasonographic image. EM: endometrium, GS: gestational sac. GS was distinct from EM at the left side of the uterus.

A 38-year-old woman, gravida 2, para 1 was admitted for a presumed ectopic pregnancy. She presented with amenorrhea and a positive urine hCG test. She had a history of a left salpingectomy for a fallopian tube pregnancy and a cesarean section. Transvaginal sonography revealed an ill-defined gestational sac and a fetal heart beat within the fundal myometrium, adjacent to the left cornua (Figure 1). We suspected a left cornual pregnancy or intramural pregnancy and therefore planned a laparoscopic approach.

**Figure 2:**
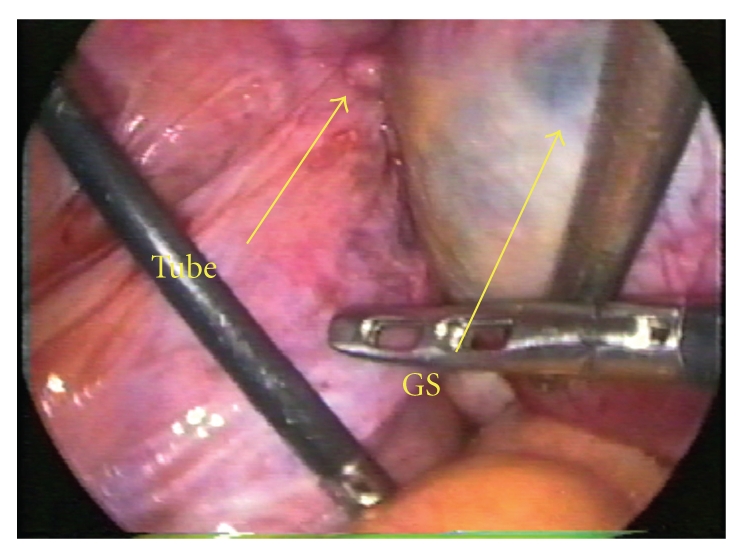
GS: gestation sac, TUBE: residual left fallopian tube. The GS was separate from residual left tube.

Laparoscopic surgery was performed with a four-port technique and carbon dioxide (CO_2_) pneumoperitoneum. Laparoscopic findings in the pelvic cavity were a uterus the size of eight-week gestation with a cystic mass 3 cm in diameter visible in the left fundal myometrium. This cyst was distinct from the residual left fallopian tube (Figure 2). The bilateral ovaries and right tube appeared normal.

**Figure 3:**
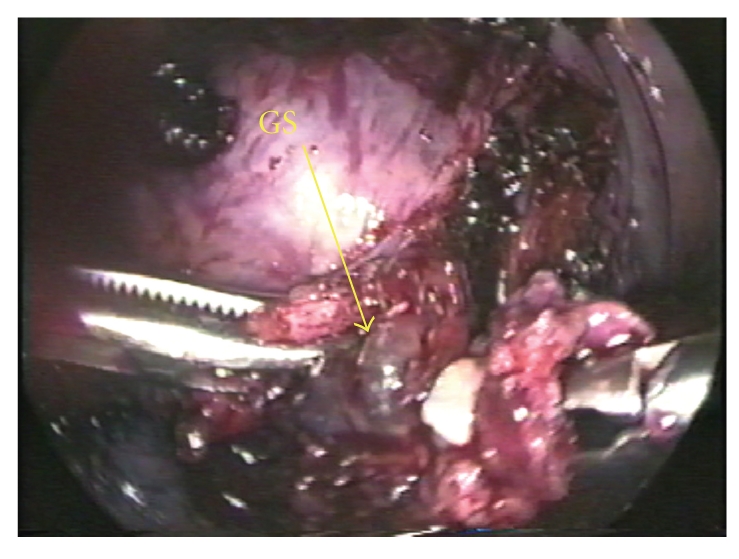
GS: gestation sac. The uterus was cut with the harmonic scalpel, and the forceps were used to grasp the GS.

**Figure 4:**
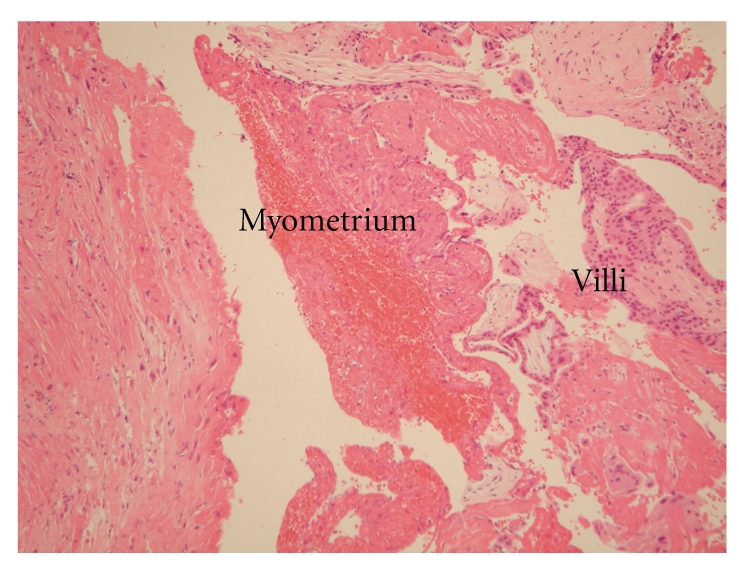
Histologic examination. Microscopic examination demonstrating villi within the myometrium and stroma.

**Figure 5:**
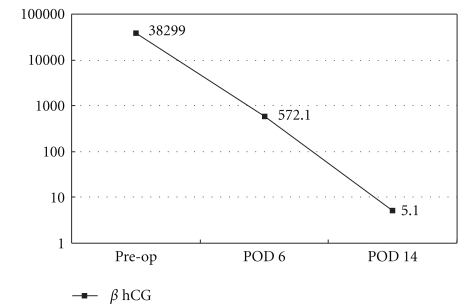
Perioperative serum beta-hCG levels. “Preop” is one day before surgery, and “POD6” is 6 days post surgery, “POD14” is 14 days post surgery. The beta-hCG dropped rapidly postoperatively.

**Figure 6:**
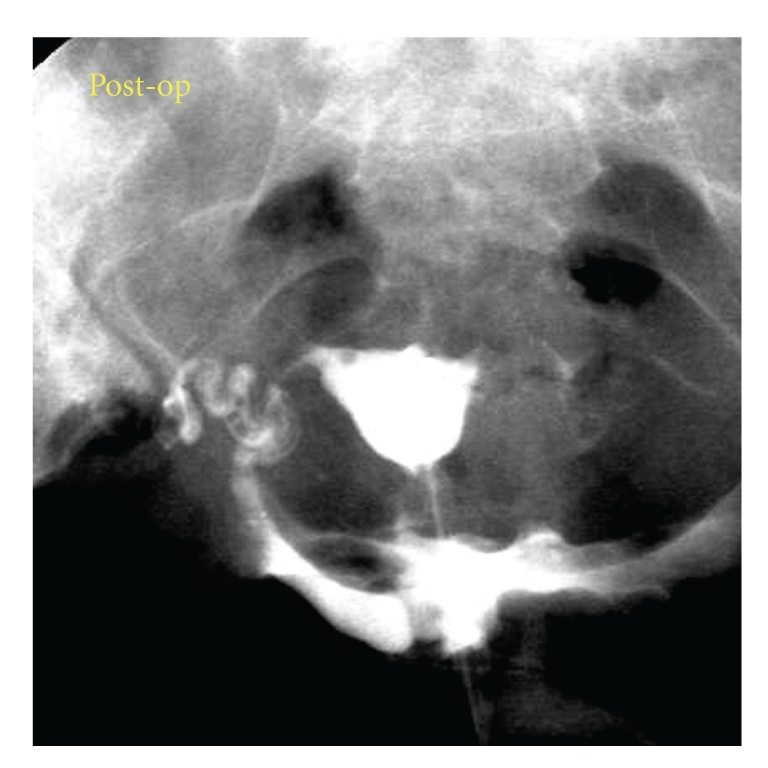
Postoperative HSG (hysterosalpingography). No filling defect is evident. Therefore the excised portion of the uterus containing the pregnancy was not one horn of a bicornuate uterus.

**Figure 7:**
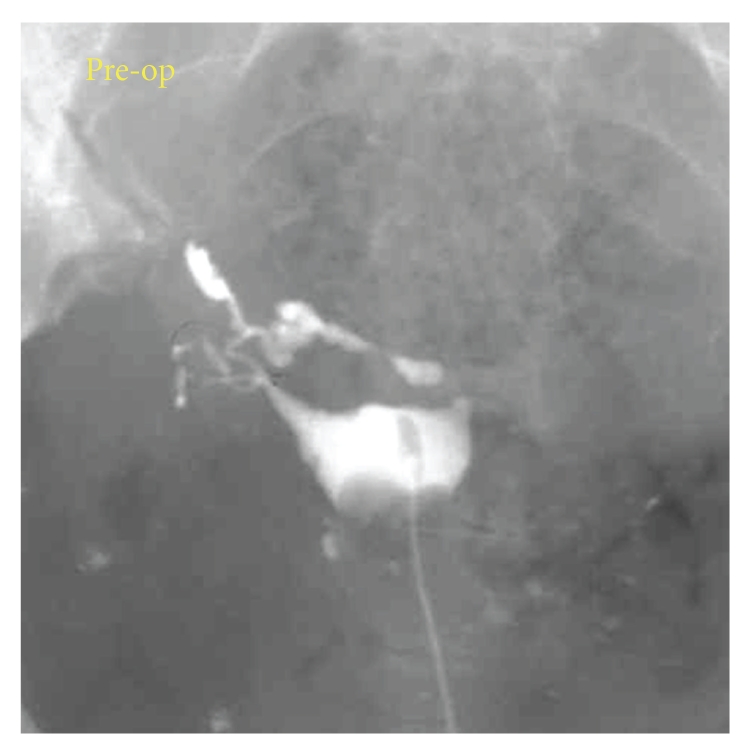
HSG prior to the pregnancy. The left tube was transected at the site of a previous ectopic pregnancy (left tubal pregnancy).

Vasopressin with physiological saline was first injected into the myometrium. The uterine serosa and myometrium were then cut with a harmonic scalpel (Ethicon Endo-Surgery, Inc., Cincinnati, OH.). Visible vessels were first coagulated with bipolar forceps prior to being cut with the harmonic scalpel. When the scalpel reached the gestational sac (Figure 3), serous fluid was released. An attempt was made to hydrodissect the gestational sac with normal saline and blunt dissect it with forceps; however, the gestational sac was inseparable. Therefore the myometrium surrounded the sac was dissected off with a grasper and the harmonic scalpel. Once the gestational sac had been excised, the site bled profusely. The myometrium was then repaired with no. 1 polyglactin Z suture and the serosa repaired in a running fashion with no. 3–0 polyglactin. Fibrin glue was used to complete hemostasis. The total operating tine was 140 minutes, and the estimated blood loss was 800 ml. The patient did not require a transfusion of any blood products. Microscopic examination confirmed the coexistence of villi, and muscle fibers. Diffuse villi and decidual tissue were present within the uterine myometrium (Figure 4). The patient's postoperative course was uneventful, and the hCG level decreased appropriately (Figure 5). The patient was discharged on postoperative day five. Followup hysterosalpingography (HSG) three months postoperatively showed no change in the appearance of the uterus (Figure 6) as compared with an HSG obtained prior to the pregnancy. Additionally, the absence of a filling defect confirmed that the tissue removed was not a horn of a bicornuate uterus (Figure 7).

## 3. Discussion

Intramural pregnancy is one of the rarest types of ectopic pregnancy [1]. More than 95% of ectopic pregnancies involve the fallopian tubes. Other sites of ectopic implantation are less frequent. We performed a systemic search of PubMed (http://www.ncbi.nlm.nih.gov/pubmed/) from 1957 to 2009. The database search used the relevant medical subject heading search (MeSH) with the term “pregnancy, ectopic” and the free query term “intramural pregnancy”. Selected subheadings were human, and English, with abstract. Thirty reports were selected, and of these articles 21 articles were case reports of an intramural pregnancy. However, there were some discrepancies in the nomenclature. Three reports were corneal [3] or interstitial pregnancies (tubal pregnancy in the broad sense) [4, 5]. Three reports were cervical pregnancy [6, 7], and one report was a subserous pregnancy at a previous myomectomy site [8]. Therefore only 17 of the retrieved cases were intramural pregnancies by the strictest definition. Abdominal hysterectomy was utilized for five cases [9–12], and a conservative abdominal procedure was performed in four cases [13–16]. Systemic or local methotrexate injection was used in three cases [17–19], and local potassium chloride injection was used for one case [20]. In one case, the gestational sac spontaneously resolved [21]. One pregnancy was continued until fetal viability was reached, and the fetus was successfully delivered by cesarean section [22]. Only one report exists describing the laparoscopic resection of an intramural pregnancy [2]. In this case, the patient went on to require a hysterectomy. Therefore, ours is the first account of the successful laparoscopic treatment of an intramural pregnancy. 

Blood flow to the gravid uterus increases significantly over baseline. Therefore, a laparoscopic procedure carries the risk of significant hemorrhage. Consequently, almost all cases of intramural pregnancy are treated with either laparotomy or medication. Historically, laparoscopy has only been used for diagnosis. Intramural pregnancies are sometimes difficult to diagnose since there may be no unusual presenting symptoms. Often, the only abnormality is sonographic. We performed a careful examination including a repeat ultrasound and determined that invasion was minimal and resection amenable to laparoscopy. We used a vasopressin solution prior to resection and fibrin glue after suturing. Finally, the surgeon was very proficient with laparoscopic suturing. These elements were essential to the successful completion of a conservative procedure.

## 4. Conclusion

We describe a case of an extremely rare form of ectopic pregnancy, which we were able to treat with conservative surgery. The intramural pregnancy is difficult to diagnose preoperatively; however, careful observation and early intervention enabled the uterus to be preserved. Laparoscopic management of such cases is feasible, provided that the operating team has advanced laparoscopic skills. Otherwise, such rare cases are to be dealt with the conventional open approach in order to minimize possible complications.

## References

[1] McGowan L (1965). Intramural pregnancy. *The Journal of the American Medical Association*.

[2] Tucker SW (1995). Laparoscopic management of an intramural pregnancy. *Journal of the American Association of Gynecologic Laparoscopists*.

[3] Gleicher N, Karande V, Rabin D, Pratt D (1994). Laparoscopic removal of twin cornual pregnancy after in vitro fertilization. *Fertility and Sterility*.

[4] Kojima E, Abe Y, Morita M, Ito M, Hirakawa S, Momose K (1990). The treatment of unruptured tubal pregnancy with intratubal methotrexate injection under laparoscopic control. *Obstetrics and Gynecology*.

[5] Okonofua FE, Ojo OS, Odunsi OA, Odesanmi WO (1990). Ectopic pregnancy associated with tubal schistosomiasis in a Nigerian woman. *International Journal of Gynecology and Obstetrics*.

[6] Neiger R, Weldon K, Means N (1998). Intramural pregnancy in a cesarean section scar: a case report. *Journal of Reproductive Medicine for the Obstetrician and Gynecologist*.

[7] Taşkin S, Taşkin EA, Cengiz B (2009). Cervical intramural ectopic pregnancy. *Fertility and Sterility*.

[8] Park WI, Jeon Y-M, Lee J-Y, Shin S-Y (2006). Subserosal pregnancy in a previous myomectomy site: a variant of intramural pregnancy. *Journal of Minimally Invasive Gynecology*.

[9] Cava EF, Russell WM (1978). Intramural pregnancy with uterine rupture: a case report. *American Journal of Obstetrics and Gynecology*.

[10] Ginsburg KA, Quereshi F, Thomas M, Snowman B (1989). Intramural ectopic pregnancy implanting in adenomyosis. *Fertility and Sterility*.

[11] Karakök M, Balat O, Sari I, Kocer NE, Erdogan R (2002). Early diagnosed intramural ectopic pregnancy associated with adenomyosis: report of an unusual case. *Clinical and Experimental Obstetrics and Gynecology*.

[12] Dousias V, Stefos T, Chouliara S, Stefanou D, Kamina S, Lolis D (2003). Intramural pregnancy with negative maternal serum b-HCG. *European Journal of Obstetrics Gynecology and Reproductive Biology*.

[13] Jin H, Zhou J, Yu Y, Dong M (2004). Intramural pregnancy: a report of 2 cases. *Journal of Reproductive Medicine for the Obstetrician and Gynecologist*.

[14] Hamilton CJCM, Legarth J, Jaroudi KA (1992). Intramural pregnancy after in vitro fertilization and embryo transfer. *Fertility and Sterility*.

[15] Hsieh Y-Y, Chang C-C, Tsai H-D, Yeh L-S, Hsu T-Y, Yang T-C (1998). Intramural pregnancy with negative maternal serum *β*-hCG: a case report. *Journal of Reproductive Medicine for the Obstetrician and Gynecologist*.

[16] Lee GSR, Hur SY, Kown I, Shin JC, Kim SP, Kim SJ (2005). Diagnosis of early intramural ectopic pregnancy. *Journal of Clinical Ultrasound*.

[17] Bhatia K, Bentick B (2004). Intramural molar pregnancy: a case report. *Journal of Reproductive Medicine for the Obstetrician and Gynecologist*.

[18] Ko H-S, Lee Y, Lee H-J (2006). Sonographic and MR findings in 2 cases of intramural pregnancy treated conservatively. *Journal of Clinical Ultrasound*.

[19] Choi D-H, Kwon H, Kim Y-S, Kim J-H (2009). Intramural pregnancy associated with adenomyosis after In vitro fertilization and embryo transfer a CASE report. *Journal of Reproductive Medicine for the Obstetrician and Gynecologist*.

[20] Khalifa Y, Redgment CJ, Yazdani N, Taranissi M, Craft IL (1994). Intramural pregnancy following difficult embryo transfer. *Human Reproduction*.

[21] Bernstein HB, Thrall MM, Clark WB (2001). Expectant management of intramural ectopic pregnancy. *Obstetrics and Gynecology*.

[22] Fait G, Goyert G, Sundareson A, Pickens A (1987). Intramural pregnancy with fetal survival: case history and discussion of etiologic factors. *Obstetrics and Gynecology*.

